# Health services restructuring in Alberta and the 2009 pandemic influenza—An untimely concurrence

**DOI:** 10.1177/0840470420909121

**Published:** 2020-03-10

**Authors:** Richard Musto, Judy MacDonald, Anne Ulrich, Kevin Fonseca

**Affiliations:** 1 O’Brien Institute of Public Health, Cumming School of Medicine, University of Calgary, Calgary, Alberta, Canada.; 2 Alberta Health Services, Calgary, Alberta, Canada.; 3 Provincial Laboratory, Alberta Precision Laboratories, Calgary, Alberta, Canada.

## Abstract

In the last 12 years, every Canadian province and territory has undertaken significant health services restructuring, with the pace of change accelerating recently. When the H1N1 Pandemic Influenza (PI) hit Alberta in the spring of 2009, the province had just begun a restructuring of health services of a scale unprecedented in Canada. The new province-wide entity, Alberta Health Services (AHS), was faced with mounting an effective response to a global communicable disease outbreak during a time of great organizational flux. In this retrospective, the authors reflect on challenges and opportunities presented during the AHS PI response related to the coordination of public health, laboratory services, emergency and disaster management, communications, and health services delivery. Lessons learned are shared that may be helpful to other provinces and territories as they continue to evolve their systems, so that they may be better prepared to respond to an untimely event such as a pandemic.

## Introduction

In the last 12 years, every province and territory in Canada has undertaken significant restructuring of their health services, with the pace of change accelerating recently.^1^ The emergence of rapidly spreading deadly infectious diseases, whether novel ones such as 2019-nCoV or familiar ones such as influenza, can be readily expected, although not their timing. When the H1N1 PI hit Alberta in the spring of 2009 (first Alberta case was confirmed on April 28), health services had just begun a restructuring of a scale unprecedented in Canada. The new, province-wide entity, Alberta Health Services (AHS), was faced with mounting an effective response to a global Communicable Disease (CD) outbreak during a time of great organizational flux.

The PI arrived in Canada just 6 years after the severe acute respiratory syndrome (SARS) did in 2003, which had exposed not only the poor level of preparedness of the health system for an outbreak of a novel agent but also how broadly disruptive it could be in a community.^2^ The SARS experience led to new investment in Public Health at the federal level, including mechanisms for better intergovernmental collaboration and information sharing. Across the country certainly, there was much attention at the local level devoted to the development of emergency response plans for infectious diseases, and Alberta was no exception. However, this continued preparedness was not prioritized in the run up to the complex restructuring of health services in Alberta in 2008-2009.^3^

Only two reviews of the Alberta experience have been published—one commissioned by the government^3^ and the other focused on surveillance.^4^ In this retrospective, the authors draw from these and their own personal recollections and reflect on challenges experienced and opportunities presented during the AHS PI response related particularly to the coordination of public health, emergency and disaster management, communications, and health services delivery. Lessons learned are shared that may be helpful to other provinces and territories as they continue to evolve their systems, so that they may be better prepared in their response to an untimely event such as a pandemic.

## The setting

In 1994, Alberta became the first province to initiate an integration of virtually all publically funded health services when it created 17 Regional Health Authorities (RHAs; Table 1). The governing boards were responsible to the provincial Ministry of Health and Wellness (AHW) but were essentially independent of one another, and the extent to which structural and/or programmatic integration was realized within the RHAs varied considerably. Some formal collaboration between authorities did occur under the initiative of various professional groups (eg, the Medical Officers of Health [MOHs], critical care, and laboratory services) that reflected both historical practice and new developments.

**Table 1.** Health services structural changes in Alberta

Year	Structural change
1994	Integration of funded health services (public health, hospitals, continuing care) into 17 regional health authorities
2000	Northern and Southern Public Health Laboratories merged
2003	Number of RHAs reduced to 9
2005	Public Health Laboratories’ governance transferred to RHAs in Edmonton and Calgary
2008	Consolidation of all RHAs, plus ground ambulance services, the Alberta Alcohol and Drug Addiction Commission, Alberta Mental Health, and the Alberta Cancer Board into Alberta Health Services

Abbreviation: RHA, Regional Health Authorities.

After a provincial election in 2008, the decision was made to further consolidate the RHAs as well as the three province-wide health service organizations plus ground ambulance services, into one province-wide service agency, Alberta Health Services, with the stated intent being to achieve more standardized, accountable and integrated services. Alberta Health Services formally began on April 1, 2009; a huge new organization with 100,000 employees, 5,500 associated physicians, a $12B budget and a new Chief Executive Officer from another country.

Only a week or two earlier, cases of a novel influenza virus had been reported in Mexico. The World Health Organization later that month declared the situation to be a Public Health Emergency of International Concern and then on June 11 declared it a pandemic influenza. Although all of the previously independent health authorities in Alberta had existing emergency preparedness plans for a pandemic, they were each at different stages of refinement. Alberta Health Services was now faced with mounting a large-scale public health response while grappling with all of the restructuring implications of becoming one provincial health organization.

## Emergency/disaster management

Alberta Health Services Emergency/Disaster (E/D) response structure for Wave I and Wave II of the H1N1 PI response looked very different. Although a provincial Emergency/Disaster Management (E/DM) program had been approved and funded early in the inception of AHS, establishment had yet to occur. Challenges arose related to the coordination of multiple legacy jurisdictions with no established incident management structure, processes, or defined roles and responsibilities that would facilitate the new organization working together. For Wave I, AHS’ internal response was reliant upon legacy RHAs’ PI preparedness (including stockpiling of PI supplies), which had been organized independently, planned differently, and were at varied levels of readiness. In the absence of an AHS Incident Management System (IMS), an ad hoc AHS Emergency Operations Centre (EOC) was established and co-located with the AHW EOC, to facilitate sharing of information, planning, decision-making, and communication required to support a coordinated, provincial Wave I response.

The inter-wave period allowed AHS to develop and implement a province-wide IMS (coordinated by the emergency disaster management team which is part of Public Health) that improved coordination and collaboration within AHS, and with government ministries, municipalities, and external agencies/industry. Alberta Health Services IMS utilized a command and control structure based on the Incident Command System (ICS) that provided a common set of principles and tools, defined roles and responsibilities, and operating procedures enabling integration and connectivity between sites, services, Zones (the five geographic subunits of AHS), external partners, and stakeholders. The AHS command and control structure included an Executive Policy Group, AHS provincial Emergency Coordination Centre, five Zone EOCs, and numerous site and service command posts, that provided strong and consistent support to operations and the frontline staff and enabled a more effective, timely and provincially collaborative management of Wave II. This AHS incident response structure stands today.

Alberta Emergency Management Agency (AEMA) Liaisons situated in provincial and municipal EOCs facilitated the sharing of information and collaborative decision-making across the province and among the lead response organizations. However, as identified in the review of the Alberta response to the PI by the Health Quality Council of Alberta, communications challenges remained both between AHS and AHW, and between central AHS leadership and the zones. Feedback provided by zone respondents was that at times the efforts to achieve consistency across the province did not take into account local nuances of geography and resources—the “one size fits all” approach chafed.^3^

The PI response experience provided an opportunity for AHS and AHW to enhance their separate and collective performance. Post PI, AHS, AHW, and AEMA developed a tripartite Alberta Pandemic Influenza Plan that aligned with the Canadian Pandemic Influenza Preparedness Planning Guidance for the Health Sector. Alberta Health Services adopted an “all hazard” approach to emergency planning and management supported by a committee structure with oversight for the development of functional operational plans that are flexible and scalable and can be adapted to any situation. This structure contributed substantially to effective responses to such later emergencies as wildfires in Slave Lake (2011) and Wood Buffalo (2016), flooding in Calgary and area (2013) as well as preparedness planning for Ebola (2014-16) and the 2015/16 Zika virus epidemic.

## Health services response

Amidst the organizational flux, pre-existing collaborations and relationships proved critical during the first wave of the response to the PI. The relationships between public health services and professionals that extended into AHW and First Nations and Inuit Health (FNIH) were relied upon heavily.

The initial case management and contact follow-up of the first PI cases by the CD units was based on existing provincial seasonal influenza guidelines. A combined team from AHW and AHS quickly developed preliminary guidance documents for the pandemic strain, including a requirement for CD professionals to collect and report individual hospitalized case data through enhanced surveillance. This ensured a standardized approach and helped to inform the development of the national pandemic guidelines. The fact of the incompatibilities of the various legacy systems of the former RHAs, including those of both public health and acute care, plus the repeated changes in official nomenclature of the pandemic strain, presented huge challenges to the team with resultant inefficiencies and gaps. An additional challenge that biased the available data toward the urban centres was the centralization of respiratory virus laboratory testing in Calgary and Edmonton, so that results from rural areas were delayed. However, the resulting situational reports were very useful in informing decision-makers and triggering interventions such as the opening of the influenza assessment centres and mass immunization clinics, plus the proactive increase in emergency and hospital staffing, and release of antiviral medications.^4^

The Provincial Laboratory for Public Health (ProvLab) was a key contributor to the surveillance activity. Prior to the pandemic, the ProvLab had been transitioning from viral culture techniques to molecular-based assays that could handle greater volumes with similar labour resources and yield reported results in a fraction of the time. These preparations were timely and valuable, as the ProvLab was the first diagnostic laboratory in Canada to provide subtype determinations for the pandemic strain, apart from the National Microbiology Laboratory.

Given their mandate to provide laboratory testing for the province for organisms that have public health implications, the ProvLab had been actively engaged in pandemic preparedness for fully a decade in conjunction with local, provincial, and national health authorities. It had stockpiled critical supplies estimated to be sufficient for a 6-month period, but with the transition to molecular-based assays and the huge volume of specimens submitted, a key infrastructure deficiency that emerged during the first wave was the need for semi-automated pipetting platforms. Funds for their purchase were slow to be approved; the equipment did not arrive in time for use during the pandemic and a high incidence of sick time among staff resulted due to repetitive strain injury from the high volume of manual pipetting required.

The availability of vaccine for wave II, while welcomed, provided another set of challenges. The pre-AHS Council of MOH, made up of all the MOHs in the province, including those from the former RHAs (and now AHS), AHW, and FNIH, worked collaboratively to provide guidance on measures to reduce the impact of the PI and liaised with community and AHS care providers to support the treatment of infected citizens. The group recommended targeting the vaccine to defined high-risk groups as consistent with those identified by the Public Health Agency of Canada; a slight modification mandated by AHW was that no one was to be denied vaccine.

Alberta had already decided to shift from a targeted to a universal program for seasonal influenza vaccination, and therefore, arrangements were in place for a greater number of community immunization clinics than in previous years. With the additional issue of the vaccine packaging (50 multi-dose vials of antigen and adjuvant each) and a complicated repackaging process, it was decided that administration would be done only by public health nurses. When it became apparent (only 4 days after the opening of the public immunization clinics) that the supply of adjuvanted vaccine was to be temporarily slowed nationally, the public health team recommended a one day closure of the clinics to permit a refocusing of the program. However, AHW made the decision to keep the clinics closed for 4 days and then reopen to a gradual progression of high-risk groups. Only quite late in the rollout of the immunization program when demand for vaccine had decreased substantially were arrangements finalized to enable pharmacists, community physicians, and paramedics and to also provide the vaccine.

Overall, the coverage rates of both high-risk groups and the general public were below that achieved in other provinces. Notable exceptions were in children younger than 5 years and First Nations individuals living on reserves. The latter achievement was in large measure due to collaborative relationships between community leaders, FNIH public health staff and local pharmacists, so that, for example, they were the first in the province to repackage the vaccine into smaller package sizes more appropriate to the settings (and incidentally, to also preposition supplies of antiviral medications in the communities).

One final example of where pre-existing relationships were so helpful comes from acute care, where the Provincial Critical Care Network was active. This group anticipated that there would be a large demand for extracorporeal membrane oxygenation life support and built upon promising research from Australia to acquire new equipment and develop treatment guidelines. They also, using a clinical database in use in Calgary, adapted a national futility rating tool to fit their experience which enabled a more aggressive approach to care in some cases. Through their established network, ventilators and respiratory therapists were deployed to several hospitals which did not otherwise have the means to provide the level of care required.

## Communications

The communications challenge and opportunity bears its own section because it permeates all aspects of the planning, response, and recovery from such a large and prolonged event. Not only was the information to be communicated changing as the pandemic evolved but also there were multiple sources available, for example, from agencies or groups in other countries. Unification of all health services under AHS clearly provided the opportunity to become the go-to authoritative source, and the manner in which the Chief MOH (within AHW) and the Senior MOH (within AHS) worked together to brief the public and health professionals was exemplary. Still, issues of timeliness, accountability, and emphasis were common at all levels and between all stakeholders, contributing to errors, inefficiencies, and missed opportunities. That AHS was the lead agency most obviously responsible for responding to the PI made it an easy target for criticism in the media, which likely contributed to a risk aversion position that extended from the political to the frontlines. For example, the early huge demand for vaccine, the expectation to avoid long clinic queues, and the evolving list of eligible risk groups all combined almost paradoxically to yield lower than expected vaccine coverage and high vaccine wastage.

## Lessons learned

There was much to be learned from Alberta’s 2009 PI experience, which may be applicable in other jurisdictions in organizational flux. Some key points are:

- Value and respect healthcare workers—they rise to the challenge.
- Establish an organization-wide E/DM program as soon as any organizational restructuring is to be implemented. An E/DM program can focus on facilitating and coordinating the functional planning for a cohesive organizational E/D response.
- Establish an organization-wide Incident Command Structure within an IMS that provides a command and control structure and processes to foster effective decision-making. It should also take into account relationships with the political levels locally and provincially.
- Establish an organization-wide E/D stockpile of supplies that are critical for each department, with accompanying processes for management, approval/release, and retrieval (where appropriate) of the stockpile holdings.
- Nurture and tap into existing collaborations and networks that cross or extend beyond the units to be amalgamated until new ones are functional.
- Develop a communication plan collaboratively with the anticipated lead agencies that assigns appropriate roles and responsibilities and respects the diversity of the various target audiences, both within and outside of the organizations.
- Develop coordinated surveillance systems across the new organization and with key partners with consistently defined data elements and triggers for intervention.

## Conclusion

The process of organizational change is difficult. The occurrence of an emergency or disaster, especially on the scale of a pandemic influenza, in the midst of such change tests an organization even further. The authors hope that this reflection will be of value to those charged with organizational preparedness for the “next big one.”
